# Developing and testing AI-based voice biomarker models to detect cognitive impairment among community dwelling adults: a cross-sectional study in Japan

**DOI:** 10.1016/j.lanwpc.2025.101598

**Published:** 2025-06-12

**Authors:** Eri Kiyoshige, Soshiro Ogata, Namhee Kwon, Yuriko Nakaoku, Chisato Hayashi, Nate Blaylock, Raymond Brueckner, Vinod Subramanian, Henry Joseph OConnell, Yusuke Yoshikawa, Kanako Teramoto, Kiyomasa Nakatsuka, Satoshi Saito, Masafumi Ihara, Misa Takegami, Kunihiro Nishimura

**Affiliations:** a Department of Preventive Medicine and Epidemiology, National Cerebral and Cardiovascular Centre, 6-1 Kishibe-Simmachi, Suita, Osaka, 564-8565, Japan; b Canary Speech, Inc., 1800 Novell Place, Suite H51, Provo, UT, 84606, USA; c Research Institute of Nursing Care for People and Community, University of Hyogo, 13-71, Kitaoji-cho, Akashi, Hyogo, 673-8588, Japan; d Department of Biostatistics, National Cerebral and Cardiovascular Centre, Suita, Osaka, 564-8565, Japan; e Department of Neurology, National Cerebral and Cardiovascular Centre, 6-1 Kishibe-Simmachi, Suita, Osaka, 564-8565, Japan; f Department of Public Health and Health Policy, School of Medicine, The University of Tokyo, 7-3-1 Hongo, Bunkyo-ku, Tokyo, 113-0033, Japan

**Keywords:** Mild cognitive impairment, Voice biomarkers, AI, Prediction model

## Abstract

**Background:**

Voice is a potential biomarker of cognitive impairment because mild cognitive impairment (MCI) can cause changes in speech patterns and tempo. Artificial intelligence (AI) can deliver voice biomarkers as prediction features, leading to a timely, noninvasive, and cost-effective detection of cognitive impairment. This study aimed to develop and test prediction models utilizing voice biomarkers to detect cognitive impairment, which AI derived from voice data of unstructured conversations in community-dwelling adults in Japan.

**Methods:**

This observational study with a cross-sectional design, included 1461 community-dwelling adults. The outcome was cognitive impairment assessed by the Memory Performance Index score from the MCI screen. Voice data was collected from 3-min open-question interviews and extracted voice biomarkers based on acoustic and prosodic features as a 512-dimensional vector of individual voice information using the voice generator, Wav2Vec2. Other considerable predictors were age, sex, and education. We developed cognitive impairment prediction models by applying the extreme gradient boosting decision tree algorithm and a deep neural network model using 979 participants. Prediction performances were tested by area under the curves (AUCs) in 482 participants who were not used for model development.

**Findings:**

We had 967 women (66·2%), 526 cognitive impairment (36·0%) participants with mean (standard deviation) age and education years of 79·5 (6·3) years old and 11·6 (2·2) years, respectively. The inclusion of voice biomarkers significantly improved AUCs (95% confidence intervals), from 0·80 (0·76, 0·84) to 0·88 (0·84, 0·91) for the age sex model and from 0·78 (0·73, 0·82) to 0·89 (0·86, 0·92) for the age sex and education model (p < 0·0001 for both comparisons by DeLong test).

**Interpretation:**

Our prediction models for cognitive impairment using voice biomarkers can provide significantly timesaving MCI screening with high prediction performances (AUC = 0·89). Voice biomarkers significantly contributed to improving prediction performance.

**Funding:**

Small Business Innovation Research (SBIR Phase 3 Fund), the Intramural Research Fund of Cardiovascular Diseases of the 10.13039/100016924National Cerebral and Cardiovascular Center, and 10.13039/501100001691JSPS KAKENHI.

**Research in context**

**Evidence before this study**

Early-detection of mild cognitive impairment (MCI), of which 92% is undiagnosed, is crucial for timely intervention for dementia due to increase by global aging. In general, MCI prediction is a more difficult task compared to dementia prediction, due to the subtlety of MCI symptoms in the transition from normal aging to dementia. Voice is a potential biomarker of MCI because it can cause changes in speech patterns and tempo. Artificial intelligence (AI) can deliver voice biomarkers as prediction features, leading to a timely, noninvasive, and cost-effective detection of MCI. On November 13th, 2023, we searched PubMed with the search terms “Deep Learning” [MeSH Terms] OR “Machine Learning” [MeSH Terms], “cognitive dysfunction” [MeSH Terms] OR “mild cognitive impairment” [All Fields], “voice” [MeSH Terms] and [All Fields], and “ROC Curve” [MeSH Terms]. We then manually collected papers related to the present study by using the keywords and referring to the searched papers by December 10th, 2024. Very few previous studies predicted MCI by using voice biomarkers derived by AI from voice data, and no previous studies tested the developed MCI prediction model in a testing dataset.

**Added value of this study**

We developed and tested AI prediction models utilizing voice biomarkers to detect cognitive impairment with high performance, which AI derived from voice data of unstructured conversations among community-dwelling adults in Japan, achieving an area under the curve (AUCs) of 0·88 (95% confidence interval [CI]: 0·84–0·91) with predictors of age, sex, and voice, and 0·89 (95% CI: 0·86–0·92) with predictors of age, sex, education, and voice. The models incorporating voice outperformed the referenced models using age and sex (AUC [95% CIs]: 0·80 [0·76, 0·84], p < 0·0001 by DeLong test) and using age, sex, and education (AUC [95% CIs]: 0·78 [0·73, 0·82], p < 0·0001 by DeLong test). Our results would be considered sufficiently valid, as the testing dataset included 487 sample sizes. This exceeds the required sample size of 310, which was calculated to be necessary for validating prediction models with an AUC of above 0·80 in the testing dataset.

**Implications of all the available evidence**

Our prediction models can substantially reduce screening time from 10 min, as required by conventional screening methods, to at least less than 1 min, enabling a timely, noninvasive, and cost-effective detection of MCI. Our use of voice biomarkers specific to acoustic and prosodic features derived from voice data of unstructured conversations allows our models to remain independent of the content of the conversation and the meaning of the words used. In addition, we believe that our prediction models using voice biomarkers can provide early detection of MCI through an easy-to administer process. This would lead to timely intervention for dementia.

## Introduction

Voice data can act as biomarkers for mild cognitive impairment (MCI), leading to a timely, non-invasive, and cost-effective detection of MCI,^1^ overcoming the current situation where 92% of expected MCI patients remain undiagnosed.^2^^,^^3^ Voice biomarkers can be derived from the voice by artificial intelligence (AI), providing a rich resource for health and diagnostic decision-making as voice features can reflect the interaction between multiple cognitive and physical processes.^4^^,^^5^ In fact, voice prediction models to detect dementia had a high prediction performance with validated area under the curve (AUC) ranging between 0·83 and 0·92.^6^, ^7^, ^8^ In the three previous studies, the reported AUCs were derived solely from voice features of the prediction models.

However, prediction models for MCI based on voice biomarkers are scarce, with the best prediction performance to date achieving a validated AUC of 0·74, indicating moderate predictive accuracy.^9^ Additionally, previous prediction models may have been influenced by the meaning of the words used in collected samples, because structured interviews were used for sample collection in their development.^7^^,^^10^, ^11^, ^12^, ^13^, ^14^ Furthermore, MCI prediction is more difficult compared to dementia prediction, due to the subtlety of MCI symptoms in the transition from normal aging to dementia. For example, previous models based on magnetic resonance imaging (MRI) results reported that a prediction performance for MCI was worse (AUC = 0·59) than that for Alzheimer's disease (AUC = 0·92) using the same methodology.^15^

Our proposed voice biomarker-based MCI prediction models, which are independent of word meaning and derived from free-talking settings with short-duration time for collecting voice data can overcome the aforementioned limitations and challenges of social implementation as seen in conventional MCI screening tools and previous MCI prediction models. Conventional paper-and-pencil screeners (e.g., Mini-Mental State Examination [MMSE] or Montreal Cognitive Assessment [MoCA]) are better at detecting overt dementia than early cognitive impairment, such as MCI.^16^, ^17^, ^18^ They require in-person administration by trained professionals and usually take approximately over 10 min.^19^^,^^20^ Such issues diminish the practical social implementation of these tools in various health settings. These limitations can be overcome by voice biomarkers.

Thus, the present study aimed to develop and test prediction models utilizing voice biomarkers to detect cognitive impairment, which AI derived from voice data of unstructured conversations in community-dwelling adults in Japan. Our model can predict cognitive impairment through a timely, non-invasive, low-cost, and an easy-to administer process, independent of the content of conversation.

## Methods

### Study design

This cross-sectional study was designed to develop prediction models applicable to a broad range of community-dwelling elderly, a population typically categorised into two subgroups: those with and those without long-term care (LTC) certification. In Japan, LTC insurance is a national insurance implemented for citizens over 65 years of age, as well as for citizens over 40 years with disabilities, under the Long-Term Care Insurance Act.^21^ To receive LTC services such as home-based nursing care and services for preventing functional limitations, a candidate must undergo a formal care needs assessment conducted by trained municipal staff using a standardised questionnaire and physician diagnosis, whereafter they are classified into one of seven care need levels (support levels 1–2, or care levels 1–5). Higher levels reflect more severe conditions that require greater levels of care. Although similar systems exist in other countries, the LTC classification in Japan is more centrally structured and nationally standardised than most, as many countries rely on locally administered or individualised assessments. To reflect the real-world diversity and heterogeneity inherent in this population, we intentionally included two distinct datasets. The first, the Kobe dataset, consisted of older adults with LTC certification who attended adult day care centres, while the second, the Nobeoka dataset, comprised older adults without LTC certification. By integrating these cohorts, we aimed to enhance the practical utility of our prediction models across varied subpopulations of community-dwelling elderly individuals. The day care center survey in Kobe city followed the same methodology as the Nobeoka survey. Both used an identical questionnaire to collect age, sex, and years of education, and employed the Japanese version of the MCI Screen (described later). Interviewers in both settings received standardised training from the official distributor of the MCI Screen, ensuring consistent administration. Supplementary Figure S1 illustrates a flow diagram of the study population.

As the first resource, we conducted cross-sectional survey with in-person cognitive assessment between March 2024 and June 2024 at adult day care service centres in Kobe city, Hyogo prefecture, Japan. We recruited participants with LTC certification who were free from speech impairment, hearing impairment, dysphonia, latent mental distress due to contents of questions, and any diagnosis of dementia. Then, 350 elderly participated in this survey.

As the second data resource, we used a cross-sectional telephone survey with agreed community-dwelling elderly without LTC certification in Nobeoka city, Miyazaki prefecture, Japan, as a secondary analysis. The telephone survey was managed and conducted by Nobeoka city as a public health service between July 2021 and December 2021 via a questionnaire. Nobeoka city sent invitation letters to all its citizens who fulfilled the following: aged between 71 and 95 years old, those without moderate to severe cognitive impairment (referring to II-A [i.e., requiring watching over] ≥ Independence in Daily Living of Elderly People with Dementia in public long-term care insurance), those without speech impairment, hearing impairment, dysphonia, or dementia, and residents living in their own house, excluding long-term hospitalisation, in Nobeoka city. From the secondary dataset of 1593 participants, 482 were excluded due to insufficient voice data for analysis (i.e., total speech duration less than 20 s) or missing demographic information (i.e., age, sex, or education). Consequently, data from 1111 older adults were included in this study.

Supplementary Figure S1 illustrates the process by which participants were divided into training and testing datasets. The Kobe and Nobeoka datasets were independently split into training and testing subsets prior to merging, thereby preventing data leakage. Specifically, in Kobe City, 350 participants from three facilities were split by facility location (178 from two facilities for training; 172 from one facility for testing) in line with the TRIPOD statement to reduce location bias.^22^ Additionally, the Nobeoka City dataset lacked residential area data due to confidentiality, so 1111 participants were randomly divided (801 for training, 310 for testing) using a 7:3 ratio. Each method was chosen based on their data characteristics and availability. Afterward, the two training datasets were merged into the combined training dataset to develop prediction models. The two testing datasets into the combined testing dataset to assess prediction performances of the developed prediction models.

### Collecting and pre-processing voice data, and extracting voice biomarkers

For details, please see Supplementary Methods. Voice data used as predictors were collected via telephone or in-person, with participants freely speaking for 3 min under minimal interviewer intervention. To mitigate recording-environment differences (e.g., telephone vs. in-person), we applied preprocessing techniques including low-pass filtering, down-sampling to 8 kHz, truncation to a maximum of 90 s, and z-normalisation. These steps ensured that differences in speech characteristics reflected true population variation rather than recording artifacts. Very short recordings (<20 s) were excluded due to insufficient information. The final audio duration ranged from 20 to 90 s (mean durations: 66 s training, 69 s testing). After exclusions for short audio length or missing demographic information, 1461 participants remained (Nobeoka: 1111; Kobe: 350).

To minimise content-dependent biases and individual speech differences seen in lexical and semantic features, we utilised the self-supervised, pre-trained Wav2Vec2 model, which is openly available and suited for extracting voice biomarkers from raw audio.^23^ The model we used was pre-trained on unlabelled LibriSpeech data and fine-tuned on its transcriptions by the original developers.^24^ Wav2Vec2 captures acoustic and phonetic context directly from raw audio without relying on explicit transcription or word prediction. Audio recordings were up-sampled from 8 kHz to 16 kHz to meet model input requirements, producing 512-dimensional embeddings representing contextualised acoustic features. These embeddings formed the sole input to our voice-only model. In the combined model, voice embeddings were concatenated with demographic variables (age, sex, education) to create a comprehensive input vector. Further technical details regarding preprocessing and Wav2Vec2 model specifics are provided in the Supplementary Appendix.

### Assessments of cognitive impairment

To asses cognitive impairment, we used the Japanese version of the MCI screen, a 10-min cognitive test consisting of immediate and delayed recall, reflecting cognitive domains, such as memory and executive function.^25^^,^^26^ The MCI screen was based on the protocol from the Consortium to Establish a Registry for Alzheimer's Disease 10-word recall test, of which the English version was originally developed by the Medical Care Corporation and endorsed by the United States Food and Drug Administration.^27^ The MCI screen calculates a Memory Performance Index (MPI) score between 0 and 100 based on Clinical Dementia Rating (CDR) scale, which distinguishes normal from cognitive impairment or MCI with a 97% accuracy.^27^^,^^28^ Japanese version of MCI screen has demonstrated minimal differences from the English version in terms of both sensitivity and specificity.^25^ Consistent with previous studies, the present study categorised the participants as having either non-cognitive impairment or cognitive impairment by using a threshold MPI score of 49·8.^29^^,^^30^ The details are presented in Supplementary Methods.

### Predictor variables other than voice data

Age, sex (as a biological variable), education years were also used as predictor variables. We treated age as a continuous variable and categorised education years into three categories (i.e., years ≤ 12, 12 < years≤ 16 years, and years> 16) corresponding to high school graduate, university graduate, and individuals with post-university education, respectively. These variables were collected via questionnaires. To improve feasibility and reduce recall bias, we used only age, sex, education years, and voice biomarkers as predictor variables. Clinical and lifestyle variables (described below) that require medical knowledge or detailed interviews were not used as predictors. Accurately collecting clinical and lifestyle variables can be time-consuming and challenging, especially for individuals without medical training. For this reason, we chose not to include these variables as predictors in our models. Instead, we focused on using only age, sex, years of education, and voice biomarkers, which can be obtained quickly and reliably without requiring detailed medical interviews.

### Demographic variables

As demographic variables for participant characteristics, we used stroke, coronary heart disease, cancer, chronic kidney disease, medication use for hypertension, diabetes, or dyslipidemia, alcohol intake, smoking, and solitude living. These variables were collected via questionnaires.

### Statistical analysis, development of prediction models, and testing their prediction performances

Characteristics of the present dataset were summarised by mean and standard deviation (SD) for continuous variables, and n and % for categorical variables, stratified by the training and testing datasets. The continuous variables were compared using the Student's t-test, and categorical variables were compared using the chi-square test. All data analyses were conducted using python 3·9 utilizing sklearn and scipy libraries and DeLong test was done using R 4·2·3.

We developed five prediction models for cognitive impairment: one reference model based on age and sex (“age sex model”); the other reference model based on age, sex, and education (“age sex education model”); one model with voice biomarkers only derived from the voice data by AI (“voice model”); another model with age and sex as well as voice biomarkers (“age sex voice model”); and the fifth model with age, sex, education, and voice biomarkers (“age sex education voice model”). The analysed dataset had no missing information in these predictors because the MCI screen collects age, sex, education years to calculate cognitive score. For missing information on the other demographic variables, please see Supplementary Table S1.

We applied an eXtreme Gradient Boosting decision tree algorithm (XGBoost) and a fully connected Deep Neural Network (DNN) model based on preliminary experiments, in which they outperformed other models (e.g., logistic regression, random forests, and support vector machine) in predictive accuracy, with acceptable computational cost. XGBoost is effective for tabular data with missing values, while DNNs can capture complex patterns in voice features. The DNN model was trained using an implementation in TensorFlow.^31^ The feature input layer was fed into the layers of a fully connected network with a ReLU activation using a binary cross-entropy loss, and then the final layer was activated by a sigmoid. The fully connected layer architecture such as layer depth, hidden layer size, and dropout rate was tuned via a hyperband search algorithm.

To address class imbalance between positive and negative samples — defined as those with and without cognitive impairment — during model training, we applied different sampling strategies depending on the model type. For XGBoost-based models, we employed bagging with under-sampling to train on balanced subsets of the data. For DNN-based models, we used oversampling via the Synthetic Minority Over-sampling Technique (SMOTE) to enhance representation of the minority class. Further methodological details are provided in the Supplementary Methods.

To select optimal hyperparameters and evaluate our DNN models, we adopted a structured approach. First, the dataset was divided into training and test sets, with the test set held out during training to prevent overfitting (Supplementary Figure S1). Second, within the training set, we employed hold-out validation, splitting data into 80% for training and 20% for validation, used to tune hyperparameters such as layer number and size. Hyperband search efficiently explored various configurations, initially training multiple models briefly, then selectively continuing only the best performers based on validation results. This iterative process gradually increased training epochs (up to 50), refining configurations each stage. The best-performing configuration was selected: the final voice and age sex voice models used one hidden layer (112 nodes) with a 0·1 dropout rate; the age sex education voice model required two hidden layers (272 and 32 nodes) with the same dropout, reflecting greater task complexity. Third, we retrained the selected models using the full original training set. Finally, we evaluated their performance on the unseen test set.

We assessed the performance of the developed prediction models by receiver operating characteristics curve area under the curve (ROC-AUC) and 95% confidence interval (CI), and accuracy, sensitivity, and specificity based on a default probability threshold of 0·5 to convert predicted probabilities into binary labels.^32^ This is a common practice in machine learning, especially when class balance is ensured, as in our case through SMOTE and under-sampling. While we explored threshold adjustment using a method similar to the Youden Index with a constraint on specificity (≥0·5), performance gains were minimal. Thus, we retained the default threshold. Higher values show better prediction performance. The 95% CIs reported in this study were estimated using the non-parametric bootstrapping method. Specifically, 1000 bootstrap resamples of the test dataset were generated with replacement, and the 2·5th and 97·5th percentiles of the resulting empirical distributions of performance metrics were used to define the CIs.

By DeLong test, we compared the age sex model vs. the voice model and the age sex voice model; we similarly compared the age sex education model vs. the age sex education voice model. We also calculated ROC-AUC (95% CI) according to the following sub-group characteristics: age as younger (<75 years old) and older (≥75 years old), sex as men and women, and education year as less than high school graduation (≤12 education years) and more than high school graduation (>12 education years).

To visualise the similarity between individuals based on all features used in the voice model, age sex voice model, and age sex education voice model, we applied supervised Uniform Manifold Approximation and Projection (UMAP) to a proximity matrix derived from voice biomarkers. UMAP is a dimensionality reduction technique that embeds high-dimensional data into a low-dimensional space while preserving local structure, enabling visualisation of complex relationships among individuals in two dimensions.

We assessed the relative importance of voice biomarkers using grouped permutation feature importance, by measuring AUC changes after masking age, sex, education, or all voice features. All 512 voice dimensions were shuffled as a group. See Supplementary Methods for details.

### Ethics approval

Ethical approval for the present study was approved by the ethics committee of the National Cerebral and Cardiovascular Centre (R21068, R20063). For the participants of the survey in adult day care service centres, we obtained written approval consent. For the participants of the survey in Nobeoka, we used an opt-out procedure. The requirement for written informed consent for the participants of the survey in Nobeoka was waived because the observed anonymised data was treated as secondary use by the present study.

### Role of the funding source

The funders of the present study had no direct or indirect role in the study design, data collection, data analysis, interpretation, or manuscript writing.

## Results

### Characteristics of participants

Among the 1461 participants, there were 967 women participants (66·2%) and 526 cognitive impairment participants (36·0%), and mean values (SD) of age and education years were, respectively, 79·5 (6·3) years old and 11·6 (2·2) years. Detailed characteristics of participants are summarised in Table 1. We compared demographic characteristics (age, sex, education) between the two data sources separately for the training and testing datasets, performing statistical tests to show demographic differences. These results, detailed in Table with accompanying p-values, to show demographic differences across data sources. In addition, we summarised the detailed characteristics of participants including demographic variables showed in Supplementary Table S2.

**Table 1:** Characteristics of the present participants.

	Training dataset			Testing dataset		
	Survey in Nobeoka	Survey in Kobe	p-value	Survey in Nobeoka	Survey in Kobe	p-value
Number of participants	801	178		310	172	
Age, mean (SD)	78·5 (5·2)	81·3 (9·0)	<0·0001	78·3 (5·1)	84·6 (6·5)	<0·0001
Education year, N (%)			<0·0001			<0·0001
≤12	674 (84·1)	123 (69·1)		269 (86·8)	121 (70·3)	
13–16	118 (14·7)	47 (26·4)		38 (12·3)	49 (28·5)	
17 and over	9 (1·1)	8 (4·5)		3 (1·0)	2 (1·2)	
Sex, N (%)			0·7585			0·0091
Men	280 (35·0)	65 (36·5)		109 (35·2)	32 (18·6)	
Women	521 (65·0)	113 (63·5)		201 (64·8)	132 (76·7)	
Cognitive impairment, N (%)	204 (25·6)	118 (66·3)	<0·0001	64 (20·8)	140 (81·4)	<0·0001

Abbreviation: SD, standard deviation.

### Performance of developed cognitive impairment models

We developed the two XGBoost-based reference models without voice biomarkers (i.e., age sex model and age sex education model) and the three DNN-based voice biomarker models to detect cognitive impairment, and showed their prediction performances in the testing dataset with better prediction performances of the voice biomarker models than those of the reference models (Table 2). Note that we reported, as the main reference models, XGBoost for the age sex and age sex education models due to the low AUCs of their DNN counterparts in the training data (Supplementary Table S3). For the voice-based models, we reported, as the main voice models, DNN, as it would better captures complex voice patterns due to the nature of DNNs.

**Table 2:** Prediction performance of all developed prediction models in testing dataset.

	XGBoost-based reference models		DNN-based voice biomarker models		
	Age sex model	Age sex education model	Voice model	Age sex voice model	Age sex education voice model
AUC –ROC (95% CI)	0·80 (0·76, 0·84)	0·78 (0·73, 0·82)	0·81 (0·77, 0·85)	0·88 (0·85, 0·91)	0·89 (0·86, 0·92)
Sensitivity (95% CI)	0·74 (0·67, 0·79)	0·68 (0·61, 0·74)	0·75 (0·69, 0·81)	0·88 (0·83, 0·92)	0·85 (0·80, 0·90)
Specificity (95% CI)	0·76 (0·71, 0·81)	0·76 (0·71, 0·81)	0·83 (0·78, 0·87)	0·75 (0·70, 0·80)	0·76 (0·71, 0·81)
Accuracy (95% CI)	0·75 (0·71, 0·79)	0·72 (0·68, 0·76)	0·79 (0·75, 0·83)	0·81 (0·78, 0·85)	0·81 (0·77, 0·84)
Positive predictive value (95% CI)	0·69 (0·63, 0·75)	0·67 (0·61, 0·74)	0·76 (0·70, 0·82)	0·72 (0·66, 0·77)	0·72 (0·67, 0·78)
Negative predictive value (95% CI)	0·80 (0·74, 0·84)	0·76 (0·71, 0·81)	0·82 (0·77, 0·87)	0·89 (0·85, 0·93)	0·87 (0·83, 0·91)

Abbreviations: ROC-AUC, receiver operating characteristic curves and area under the curves; CI, confidence interval; DNN, Deep Neural Network; XGBoost, eXtreme Gradient Boosting decision tree algorithm.

CIs were obtained from computing 1000 bootstrap sets.

**Fig. 1:**
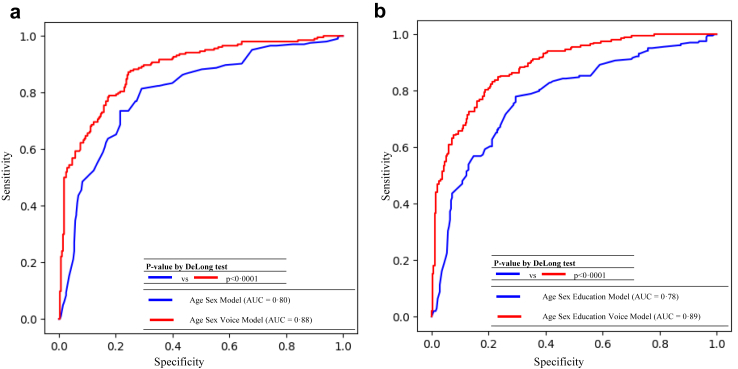
**Receiver operating characteristic curves and their AUCs for prediction performance of the developed cognitive impairment prediction models in the testing dataset**. Abbreviation: AUC, Area under the curves. (a) age sex model vs. age sex voice model and (b) age sex education model vs. age sex education voice model.

Adding voice biomarkers to the two reference models significantly improved ROC-AUCs in the testing dataset. Specifically, ROC-AUCs (95% CIs) increased from 0·80 (0·76, 0·84) in the age sex model based on XGBoost to 0·88 (0·84, 0·91) in the age sex voice model based on DNN (p < 0·0001 by DeLong test, shown in Fig. 1a), and increased from 0·78 (0·73, 0·82) in the age sex education model based on XGBoost to 0·89 (0·86, 0·92) in the age sex education voice model based on DNN (p < 0·0001 by DeLong test, shown in Fig. 1b). A similar significant increase in AUC (95% CI) was observed even when comparing between the three DNN-based models including voice biomarkers, and the DNN-based age sex model with AUC of 0·71 (0·66, 0·76) and age sex education model with AUC of 0·67 (0·62, 0·72), by p < 0·0001 of DeLong test (Supplementary Table S3 and Supplementary Figure S2). The prediction performances of the five models in the training dataset are shown in Supplementary Table S3.

In addition to these high AUCs, the age sex voice model and the age sex education voice model had higher sensitivity while maintaining similar high specificity, compared with the two reference models without voice biomarkers (sensitivity [95% CI]: 0·88 [0·83, 0·92] for age sex voice model vs. 0·74 [0·67, 0·79] in age sex model; 0·85 [0·80, 0·90] for age sex education voice model vs. 0·68 [0·61, 0·74] in age sex education model in Table 2 and Supplementary Table S3).

### Sub-group performance for prediction model

**Fig. 2:**
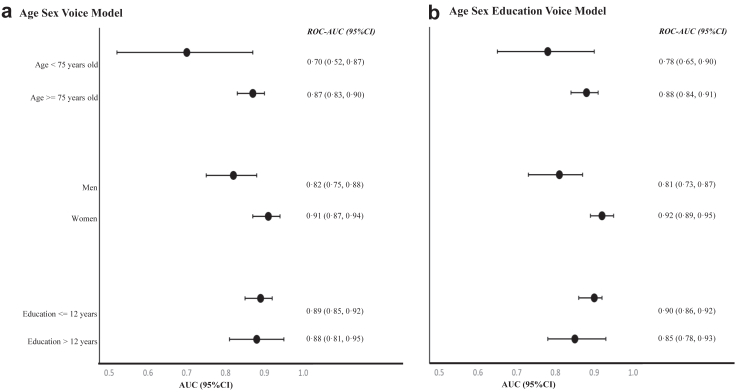
**AUCs in sub-groups according to age, sex, or education for age sex voice model and age sex education voice model in the testing dataset**. Abbreviations: AUC, Area under the curves; CI, confidence interval. (a) age sex voice model and (b) age sex education voice model. CIs were obtained from computing 1000 bootstrap sets.

For age sex voice model and age sex education voice model, we showed ROC-AUCs (95% CIs) in the sub-groups by age, sex, or education in the testing dataset in Fig. 2 and Supplementary Table S4. High prediction performance of the two models was observed in almost all sub-groups with AUCs ranging from 0·78 to 0·92, except the age sex voice model for the sub-group aged <75 years old with an AUC of 0·70.

### Visualisation of similarities in predictor variables

**Fig. 3:**
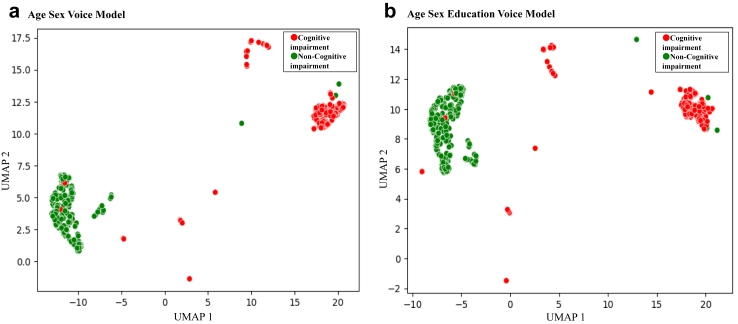
**Two-dimensional visualisations of predictor variables from the proximity matrix of all participants using supervised UMAP, coloured by cognitive impairment and healthy observed status, in the testing data**. Abbreviation: UMAP, uniform a method of manifold approximation and projection. (a) age sex voice model and (b) age sex education voice model. Red dots show cognitive impairment individuals, and green dots show non-cognitive impairment individuals.

The supervised UMAP visualisation found two clusters based on similarities in predictor variables used by each of the prediction models in the testing dataset. Additionally, we coloured the dots (corresponding to each participant) based on the observed cognitive impairment categories (Fig. 3 and Supplementary Figure S3). One cluster group mainly consisted of the cognitive impairment group; in contrast, the other mainly consisted of non-cognitive impairment. These results showed the predictor variables of age, sex, education years, and voice biomarkers were able to divide the participants into cognitive impairment and non-cognitive impairment.

### Relative importance of voice biomarkers

Grouped permutation feature importance analysis revealed that voice biomarkers had the largest impact on model performance (Supplementary Figure S4). In the model including age, sex, education, and voice biomarkers, permuting all 512 voice dimensions reduced AUC from 0·89 to 0·65, indicating a substantial drop of 0·24. In comparison, masking age led to a smaller AUC decrease of 0·12 (from 0·89 to 0·77), while masking sex and education had minimal effects (AUCs: 0·88 and 0·87, respectively; drops of 0·01 and 0·02).

## Discussion

We developed cognitive impairment prediction models using voice biomarkers and showed high prediction performances of those models in the testing dataset (AUC [95% CI]: 0·88 [0·84, 0·91] for age sex voice model and 0·89 [0·86, 0·92] for age sex education voice model). Additionally, adding voice biomarkers to the two reference models without voice biomarkers significantly improved prediction performance to classify cognitive impairment or non-cognitive impairment (p-value for DeLong test <0·0001). Our models were validated by using a sufficient sample size to ensure reliable performance. AUCs according to subgroups of age, sex, and education were high for age sex voice model and age sex education voice model in almost all sub-groups with AUCs ranging from 0·78 to 0·92, except the age sex voice model for the sub-group aged <75 years old with an AUC of 0·70. Since individuals with dementia were excluded from our study population, the current prediction cognitive impairment can be regarded as approximating the prediction of MCI. Therefore, social implementation of our voice and reference models can offer early-detection of MCI through a timely, non-invasive, low-cost, and an easy-to-administer process, leading to timely intervention for dementia. This is because our voice biomarker models utilise AI-derived voice biomarkers from easily collected voice data of unstructured conversations.

Our best prediction model using age, sex, education, and voice biomarkers to detect cognitive impairment had high ROC-AUC (95% CI) of 0·89 (0·86, 0·92), which was developed in the training dataset (N = 979) and tested in the testing dataset (N = 482). A previous prediction model using voice biomarkers to detect MCI had ROC-AUC of 0·74 assessed by 10-fold cross-validation in community-dwelling participants from Framingham Heart Study (N = 1084 for developing the model with the 10-fold cross-validation to get the reported prediction performance).^9^ Previous prediction models using voice biomarkers to detect dementia in a systematic review were usually developed in small sample sizes (i.e., N for developing the model with cross-validation to get the reported prediction performance <500), as seen in approximately twenty papers.^6^ Moreover, five studies on predicting dementia, including three studies from systematic reviews, reported AUCs of 0·89 (N in testing dataset = 24) in testing datasets^33^ and the AUCs obtained by cross-validation ranged from 0·83 to 0·92, based on datasets where the number of participants used to develop the models was between 30 and 150.^6^, ^7^, ^8^ In general, MCI prediction is a more difficult task compared to dementia prediction, due to the subtlety of MCI symptoms in the transition from normal aging to dementia.

Voice data is useful in predicting cognitive impairment including MCI and dementia based on the present and previous results. Adding voice biomarkers to the reference models without voice biomarkers significantly improved MCI prediction performance (ROC-AUC [95% CI]: 0·88 [0·84, 0·91] for age sex voice model vs. 0·80 [0·76, 0·84] for age sex model with p-value for DeLong test <0·0001; 0·89 [0·86, 0·92] for age sex education voice model vs. 0·80 [0·73, 0·82] for age sex education model with p-value for DeLong test <0·0001) in the present study. Additionally, in the present study, grouped permutation analysis showed the strong predictive value of voice features in the age sex education voice model for cognitive impairment, surpassing traditional demographic variables (i.e., age, sex, and education). Furthermore, the present prediction models solely using voice biomarkers without age and sex had ROC-AUCs of 0·81 (0·77, 0·85) in the testing dataset. Those ROC-AUCs were higher than the threshold of 0·80, which is typically required for sufficient disease discrimination in clinical settings.^34^ The prediction models using voice biomarkers to detect dementia had AUCs ranging between 0·83 and 0·92,^6^, ^7^, ^8^ which shows a more severe cognitive impairment than MCI. Most of these AUCs were higher than prediction models for dementia without voice biomarkers of which AUCs ranged between 0·72 and 0·89, reported in a systematic review of validated AUCs in seven studies based on elderly participants.^35^

Age and education are stable risk factors for cognitive decline, with education linked to delayed onset.^36^ This supports consistent model performance.^37^ Our reference model using age, sex, and education showed good predictive performance (AUC = 0·78; 95% CI: 0·73–0·82), consistent with a prior dementia model (C-statistic = 0·78; 95% CI: 0·76–0·81).^37^ Similarly, our age sex education voice model achieved an AUC of 0·78. In contrast, the age–sex–voice model performed poorly in those aged <75 years (AUC = 0·70), further supporting the value of including education.

The present study had strengths. First, our prediction models can substantially reduce screening time from 10 min, as required by conventional screening methods, to approximately 1 min of voice data. This refers to the average amount of voice input needed when applying our developed models in practical or clinical settings. The model utilise acoustic and prosodic features derived from voice data of unstructured conversations. This enables our models to remain independent of the content of conversation and the meaning of the words used. Second, our sample size of 487 in the testing dataset was sufficient to validate prediction models.^38^ This was because a sample size of 310 for testing datasets is required to validate prediction models with AUC above 0·80 over (for details, see Supplementary Methods).^38^

Our study had several limitations. First, we did not conduct prospective validation to evaluate the generalisability of the present prediction models.^39^ However, our testing dataset had sufficient sample size to evaluate prediction models with AUC of 0·80 or over.^38^ Second, the proposed AI-based models have inherent “black box” characteristics, as the voice features are extracted using a pretrained Wav2Vec2 model. These 512-dimensional embeddings capture linguistic and prosodic information, but their individual dimensions are not directly interpretable, as is typical with deep learning representations. Therefore, the models should be used as a screening tool, not a standalone diagnostic method, and must be complemented by clinical assessment. Third, the present study defined cognitive impairment solely based on memory test performance (i.e., the MPI score from the MCI Screen), without broader cognitive or biomarker data. This may reduce precision, increase heterogeneity within the MCI/dementia group, and limit the model's utility for early risk screening. However, the MPI score has been reported to distinguish suspected MCI from normal ageing with 97% accuracy.^25^ Finally, although demographic differences—such as age, sex, education, and cognitive status—existed between the two datasets, these demographic variables were explicitly accounted for in our predictive models. Subgroup analyses, in which the models were tested separately within each dataset, demonstrated consistent and robust model performance within each subgroup individually, suggesting the model's stable predictive accuracy across community-dwelling elderly with and without long-term care certification. Specifically, the models achieved relatively high AUCs in the source-specific test sets (0·80 [95% CI: 0·74, 0·85] in Nobeoka and 0·80 [95% CI: 0·71, 0·88] in Kobe for the age sex voice model; 0·80 [95% CI: 0·74, 0·84] in Nobeoka and 0·79 [95% CI: 0·70, 0·87] in Kobe for the age sex education voice model). The higher AUC in the combined test dataset (The Kobe and Nobeoka datasets) of 0·88 (95% CI: 0·85, 0·91) for the age sex voice model and of 0·89 (0·86, 0·92) for the age sex education voice model may reflect integration-related factors such as sample size and class separability. Notably, the observed percentage of cognitive impairment in this study appears high (36·0%), which is likely attributable to the advanced age of the present study population (mean values [SD] of age: 79·5 [6·3] years old). Although cognitive impairment is common among the elderly, this age profile may not fully reflect real-world community-dwelling elderly populations. In addition, the present study population is exclusively of Japanese ethnicity, which limits applicability to other racial or ethnic populations. Future validation studies may be warranted to assess the generalisability of our findings.

### Conclusions

We developed and tested cognitive impairment prediction models using voice biomarkers with a high prediction performance (AUC [95% CI] = 0·89 [0·86, 0·92]). Additionally, adding voice biomarkers to the reference models without voice biomarkers significantly improved the prediction performance. We believe that our cognitive impairment prediction models using voice biomarkers can reduce screening time for MCI and offer early detection of MCI through a timely, non-interventive, low-cost, and an easy-to administer process. This would result in a timely intervention for dementia.

## Contributors

EK, SO, MT, YN, KN (Kunihiro Nishimura): study design; SO, KN (Kunihiro Nishimura): funding; EK, SO, MT, YN, SS, MI, CH, KN (Kunihiro Nishimura): data collection; EK, NK: data analysis; EK, SO, NK, NB: data interpretation and writing; EK, SO, NK, NB, KN (Kunihiro Nishimura): had full access to all the data and attest to the completeness and accuracy of the data and data analyses; All authors, EK, SO, NK, YN, CH, NB, RB, VS, HO, YY, KT, KN (Kiyomasa Nakatsuka), SS, MI, MT, KN (Kunihiro Nishimura): critical review of the manuscript, and read and approved the final manuscript.

## Data sharing statement

The data developing the present prediction models are not publicly available to protect the privacy of a patient, as the granularity of the data may result in re-identification.

## Declaration of interests

We have no conflicts of interest to declare.

## Appendix A Supplementary data

Supplementary Figures and Tables

Translated abstract
