# Supplementary material for: Developing and testing AI-based voice biomarker models to detect cognitive impairment among community dwelling adults: a cross-sectional study in Japan
Source: Lancet Reg Health West Pac. 2025 Jun 12;59:101598. doi: 10.1016/j.lanwpc.2025.101598 (PMC12266181; doi:10.1016/j.lanwpc.2025.101598)
Supplement: Supplementary Figures and Tables [file mmc1.docx]

**Supplementary Files**

**Table of Contents**

[Supplementary Methods 2](#_Toc199154182)

[Supplementary Table S1. 5](#_Toc199154183)

[Supplementary Table S2. 6](#_Toc199154184)

[Supplementary Table S3. 7](#_Toc199154185)

[Supplementary Table S4. 8](#_Toc199154186)

[Supplemental Figure S1. 9](#_Toc199154187)

[Supplemental Figure S2. 10](#_Toc199154188)

[Supplementary Figure S3. 11](#_Toc199154189)

[Supplementary Figure S4. 12](#_Toc199154190)

# **Supplementary Methods**

***Collecting and Pre-Processing Voice Data, and Extracting Voice Biomarkers***

Voice data, used as a predictor in the present study, was collected via telephone and in-person and consisted only of the participants’ voice. The participants were asked to speak freely for 3 minutes with minimum interviewer intervention. To reduce variability arising from differences in recording environments including telephone vs in-person, we applied preprocessing techniques including low-pass filtering, down-sampling to 8 kHz, truncation of recordings to 90 seconds, and individual audio sample z-normalisation. These steps ensured that observed differences represent population characteristics rather than recording artifacts. Specifically, we collected audio files in the uncompressed WAV format with a sampling rate of 8 kHz, the standard for phone calls, in the survey in Nobeoka, and 44·1 kHz down-sampled to 8kHz in the survey in the adult day care service centres in Kobe, matching the sampling rate of the phone calls, to reduce any potential negative effects of compression on the final prediction of our models. The length of audio samples varied across speakers in the datasets. For example, very short speech samples did not contain sufficient information to robustly detect cognitive impairment from speech; samples with voice activity duration shorter than 20 seconds were removed from the data set, as described above. In addition, speech samples exceeding 90 seconds were truncated, based on the distribution of available audio lengths, to standardise the data and reduce variability between recordings. The 90-second cutoff was chosen because it would capture most meaningful speech content while avoiding the long-tail effects of exceptionally long recordings. This decision resulted in a final range of 20 to 90 seconds, with an average duration of 66 seconds for training samples and 69 seconds for testing samples. Of the 1,943 (1,593 in Nobeoka plus 350 in Kobe) participants from whom samples were collected, 482 participants who had short audio files or missing information on age, sex, and education years were excluded, leaving a final total of 1,461 (1,111 in Nobeoka + 350 in Kobe) participants whose samples were included in our study.

In the present study, we focused on the signal-based representation that was less dependent on speech topics or contents. Previous studies had frequently employed lexical and semantic features as voice biomarkers by analysing and defining speech patterns of people who suffered from cognitive impairment. However, the individual differences in speech made it challenging to generalise the relationship between speech and cognitive function. Further, the content-based hand-engineered biomarkers potentially contained limitations in the applications.

To address these limitations and to make our cognitive impairment models, we used a pre-trained Wav2Vec2 model, which is openly available and suited for extracting voice biomarkers from raw audio.^1^ The model we used was pre-trained on unlabelled LibriSpeech data and fine-tuned on its transcriptions by the original developers.^2^ We did not apply further fine-tuning. Instead, we used it solely as a feature extractor. Wav2Vec2 is a self-supervised learning framework designed to learn speech representations from large-scale, unlabelled audio data. Rather than relying on transcriptions or labels, it is trained to predict masked segments of raw audio based on their surrounding context. This enables the model to capture rich, contextualised acoustic and phonetic features directly from the waveform in a latent space, without reconstructing the original signal or predicting words. The version of Wav2Vec2 we used was pre-trained and fine-tuned on 960 hours of 16kHz speech from the LibriSpeech dataset.^3^

As our original recordings were sampled at 8kHz, we up-sampled them to 16kHz to meet the model’s input requirements. We then passed the audio through the Wav2Vec2 model and extracted 512-dimensional embeddings from its final layer. These dense vectors encode rich contextual speech features and served as the sole input to our voice-only model for cognitive impairment classification. In the combined model using both voice and demographic variables (age, sex, education), we concatenated the 512-dimensional voice embeddings with these three variables, yielding a 515-dimensional input vector. Because Wav2Vec2 representations are learned from extensive unlabelled speech, they capture a wide range of acoustic and lexical characteristics, making them highly effective for deriving voice biomarkers relevant to cognitive status.

***MCI screen***The MCI Screen is a brief, approximately 10-minute cognitive assessment developed to detect Mild Cognitive Impairment (MCI), a condition that may precede Alzheimer’s disease. The Japanese version of the MCI Screen includes tasks that evaluate immediate and delayed recall, which together assess key cognitive domains such as memory and executive function.^4,5^ The test is derived from the Consortium to Establish a Registry for Alzheimer’s Disease (CERAD) 10-word recall test and was originally developed in English by the Medical Care Corporation, receiving endorsement from the U.S. Food and Drug Administration.

The primary outcome of the MCI Screen is the Memory Performance Index (MPI), a score ranging from 0 to 100 that quantifies memory performance and based on the Clinical Dementia Rating (CDR) scale. Higher scores indicate better cognitive functioning, which distinguishes normal from cognitive impairment or MCI with a 97% accuracy.^6,7^ Japanese version of MCI screen has demonstrated minimal differences from the English version in terms of both sensitivity and specificity.^4^ Consistent with previous studies, the present study categorised the participants as having either non-cognitive impairment or cognitive impairment by using a threshold MPI score of 49.8.^8,9^ Since individuals with dementia were excluded from our study population, the current prediction cognitive impairment can be regarded as approximating the prediction of MCI. Importantly, the MPI is adjusted for factors such as age, sex, and educational level, ensuring the score’s applicability across a diverse population.

As a tool, the MCI Screen enables early detection of memory decline and provides clinicians with a standardised and validated method for identifying individuals at risk for dementia. It is also useful for monitoring cognitive changes over time or evaluating the effectiveness of therapeutic interventions.

***Handling of Class Imbalance***

To address class imbalance between positive and negative samples — defined as those with and without cognitive impairment — during model training, we adopted different sampling strategies depending on the prediction model as follows.

For the eXtreme Gradient Boosting decision tree algorithm (XGBoost)-based models, we applied bagging with under-sampling.^10^ To address class imbalance, we created multiple balanced training subsets, each containing an equal number of samples from the majority and minority classes. Separate models were trained on each subset (n = 10 models), and their outputs were aggregated via bagging to obtain the final prediction. This approach helps mitigate overfitting, ensures that the model learns from the entire dataset across subsets, and maintains class balance within each training batch.

For the deep neural network (DNN) algorithm-based models, we used oversampling to correct class imbalance. Bagging with under-sampling was not applied to these models because we needed to reduce training time and simplify implementation considering that the DNN algorithm generally takes longer time than the XGBoost algorithm. We employed the Synthetic Minority Over-sampling Technique (SMOTE), which generates synthetic samples by interpolating between existing minority class instances and their k-nearest neighbors in the feature space.^11^ This method increases the diversity and representation of the minority class, thereby improving model robustness and balance during training.

***Grouped Permutation Feature Importance Analysis***

We employed grouped permutation feature importance to estimate the collective contribution of voice biomarkers to model performance,^12^ AUC. Due to the high dimensionality of the Wav2Vec2-derived voice biomarkers (512 dimensions), evaluation of individual biomarkers using standard permutation or SHapley Additive exPlanations (SHAP) methods was not appropriate. Instead, we permuted the entire set of voice biomarkers together as a group to assess their impact on the AUC of the age sex voice model and age sex education voice model. We performed this analysis on the model using age, sex, education years, or voice biomarkers as input. The performance drop in AUC after shuffling (masking) each variable group (voice biomarkers, age, sex, and education) was used to infer relative importance.

***Sample size***
We determined the minimum sample size of 310 based on a method proposed by Riley et al. (BMJ, 2024)^13^, which provides a framework for calculating the required sample size for testing studies based on the anticipated AUC.

We assumed an AUC of 0.81, as an AUC greater than 0.8 is generally considered to indicate good discriminatory ability for clinical decision-making.^14^ For the calculation, we also assumed a cognitive impairment prevalence of 40%, based on population characteristics. Specifically, in our training dataset, the mean age was approximately 80 years. Previous studies have reported a prevalence of mild cognitive impairment (MCI) of 30.2% among community-dwelling adults aged 80–85 years,^15^ and 44.5% among those with a mean age of 60.7 years.^16^ Our test dataset includes individuals with long-term care certification, who are at higher risk for cognitive decline.

Based on these assumptions, the sample size calculation indicated that at least 301 participants were needed to reliably estimate an AUC of 0.81. Therefore, we set the minimum sample size at 310 to ensure sufficient statistical power for validation.

*Reference*

1 Baevski A, Zhou H, Mohamed A, Auli M. wav2vec 2.0: A Framework for Self-Supervised Learning of Speech Representations. *Adv Neural Inf Process Syst* 2020; **2020**-**December**. https://arxiv.org/abs/2006.11477v3 (accessed Dec 23, 2024).

2 wav2vec. https://github.com/facebookresearch/fairseq/tree/main/examples/wav2vec#wav2vec-20 (accessed April 11, 2025).

3 Panayotov V, Chen G, Povey D, Khudanpur S. Librispeech: An ASR corpus based on public domain audio books. *ICASSP, IEEE Int Conf Acoust Speech Signal Process - Proc* 2015; **2015**-**August**: 5206–10.

4 Cho A, Sugimura M, Nakano S, Yamada T. The Japanese MCI screen for early detection of Alzheimer’s disease and related disorders. *Am J Alzheimers Dis Other Demen* 2008; **23**: 162–6.

5 THE MCI SCREEN A Pragmatic Clinical Tool for Assessing Memory Concerns in a Primary Care Setting. https://www.google.com/search?q=THE+MCI+SCREEN+A+Pragmatic+Clinical+Tool+for+Assessing+Memory+Concerns+in+a+Primary+Care+Setting (accessed April 11, 2025).

6 Shankle WR, Mangrola T, Chan T, Hara J. Development and validation of the Memory Performance Index: reducing measurement error in recall tests. *Alzheimers Dement* 2009; **5**: 295–306.

7 Shankle WR, Romney AK, Rara J, *et al.* Methods to improve the detection of mild cognitive impairment. *Proc Natl Acad Sci U S A* 2005; **102**: 4919–24.

8 Yamamoto K, Shiota S, Yoshiiwa A, Chishima T, Takigami S, Miyazaki E. Cognitive Function and Olfactory Impairment in Community-Dwelling Older Adults Attending a Salon. *J Prim Care Community Health* 2022; **13**. DOI:10.1177/21501319221117793.

9 Nogi S, Uchida K, Maruta J, *et al.* Utility of olfactory identification test for screening of cognitive dysfunction in community-dwelling older adults. *PeerJ* 2021; **9**. DOI:10.7717/PEERJ.12656.

10 Wallace BC, Small K, Brodley CE, Trikalinos TA. Class Imbalance, Redux. *2011 IEEE 11th Int Conf Data Min* 2011; : 754–63.

11 Chawla N V, Bowyer KW, Hall LO, Kegelmeyer WP. SMOTE: Synthetic Minority Over-sampling Technique. *J Artif Intell Res* 2002; **16**: 321–57.

12 Au Q, Herbinger J, Stachl C, Bischl B, Casalicchio G. Grouped feature importance and combined features effect plot. *Data Min Knowl Discov* 2022; **36**: 1401–1450.

13 Riley RD, Snell KIE, Archer L, *et al.* Evaluation of clinical prediction models (part 3): calculating the sample size required for an external validation study. *BMJ* 2024; **384**. DOI:10.1136/BMJ-2023-074821.

14 Mandrekar JN. Receiver operating characteristic curve in diagnostic test assessment. *J Thorac Oncol* 2010; **5**: 1315–6.

15 Ninomiya T, Nakaji S, Maeda T, *et al.* Study design and baseline characteristics of a population-based prospective cohort study of dementia in Japan: the Japan Prospective Studies Collaboration for Aging and Dementia (JPSC-AD). *Environ Health Prev Med* 2020; **25**: 64.

16 Miyake Y, Tanaka K, Senba H, *et al.* Hearing Impairment and Prevalence of Mild Cognitive Impairment in Japan: Baseline Data From the Aidai Cohort Study in Yawatahama and Uchiko. *Ear Hear* 2020; **41**: 254–8.

# **Supplementary Table S1.**

**Proportions of missing values by data resources, training and testing datasets**

|  | Training dataset | |  | Testing dataset | |
| --- | --- | --- | --- | --- | --- |
|  | Survey in Nobeoka | Survey in Kobe^1^ |  | Survey in Nobeoka | Survey in Kobe^1^ |
| Number of participants | 801 | 178 |  | 310 | 172 |
| Age, N(%) | 0 (0·0) | 0 (0·0) |  | 0 (0·0) | 0 (0·0) |
| Sex, N(%) | 0 (0·0) | 0 (0·0) |  | 0 (0·0) | 0 (0·0) |
| Education, N(%) | 0 (0·0) | 0 (0·0) |  | 0 (0·0) | 0 (0·0) |
| Stroke, N (%) | 21 (2·6) | 0 (0·0) |  | 13 (4·2) | 0 (0·0) |
| Coronary Heart Disease, N (%) | 22 (2·7) | 0 (0·0) |  | 15 (4·8) | 0 (0·0) |
| Cancer, N (%) | 16 (2·0) | 0 (0·0) |  | 12 (3·9) | 0 (0·0) |
| Chronic Kidney Disease, N (%) | 19 (2·4) | 0 (0·0) |  | 13 (4·2) | 0 (0·0) |
| Medication use for Hypertension, N (%) | 20 (2·5) | 0 (0·0) |  | 11 (3·5) | 0 (0·0) |
| Medication use for Diabetes, N (%) | 37 (4·6) | 0 (0·0) |  | 19 (6·1) | 0 (0·0) |
| Medication use for Dyslipidemia, N (%) | 35 (4·4) | 0 (0·0) |  | 11 (3·5) | 0 (0·0) |
| Alcohol habit, N (%) | 13 (1·6) | 0 (0·0) |  | 9 (2·9) | 0 (0·0) |
| Smoking habit, N (%) | 13 (1·6) | 0 (0·0) |  | 10 (3·2) | 0 (0·0) |
| Living alone, N (%) | 11 (1·4) | 0 (0·0) |  | 8 (2·6) | 0 (0·0) |

1 In a survey conducted in Kobe, investigators checked whether participants had forgotten to respond to the questionnaire. When they found cases where participants had overlooked questions, they reminded them verbally to complete their responses.

# **Supplementary Table S2.**

**Characteristics of present participants by cognitive impairment group, data resources, and training and testing datasets.**

|  | **Training dataset** | | | | |  | **Testing dataset** | | | | |
| --- | --- | --- | --- | --- | --- | --- | --- | --- | --- | --- | --- |
|  | **Survey in Nobeoka** | |  | **Survey in Kobe** | |  | **Survey in Nobeoka** | |  | **Survey in Kobe** | |
|  | **non-Cognitive Impairment** | **Cognitive Impairment** | | **non-Cognitive Impairment** | **Cognitive Impairment** | | **non-Cognitive Impairment** | **Cognitive Impairment** | | **non-Cognitive Impairment** | **Cognitive Impairment** |
| Number of participants | 598 | 203 |  | 60 | 118 |  | 246 | 64 |  | 32 | 140 |
| Age, mean (SD) | 77·2 (11·2) | 77·4 (4·6) |  | 83·3 (6·8) | 81·7 (5·3) |  | 77·3 (4·7) | 82·3 (4·6) |  | 79·1 (8·0) | 85·9 (5·4) |
| Education level, N (%) |  |  |  |  |  |  |  |  |  |  |  |
| <= 12 | 36 (60·0) | 490 (81·9) |  | 87 (73·7) | 184 (90·6) |  | 211 (85·8) | 58 (90·6) |  | 17 (53·1) | 104 (74·3) |
| 13-16 | 19 (31·7) | 103 (17·2) |  | 28 (23·7) | 15 (7·4) |  | 32 (13·0) | 6 (9·4) |  | 14 (43·8) | 35 (25·0) |
| 17+ | 5 (8·3) | 5 (0·8) |  | 3 (2·5) | 4 (2·0) |  | 3 (1·2) | 0 (0·0) |  | 1 (3·1) | 1 (0·7) |
| Gender, N (%) |  |  |  |  |  |  |  |  |  |  |  |
| Male | 18 (30·0) | 186 (31·1) |  | 47 (39·8) | 94 (46·3) |  | 76 (30·9) | 33 (51·6) |  | 9 (28·1) | 31 (22·1) |
| Female | 412 (68·9) | 109 (53·7) |  | 42 (70·0) | 71 (60·2) |  | 170 (69·1) | 31 (48·4) |  | 23 (71·9) | 109 (77·9) |
| Stroke, N (%) | 20 (33·3) | 42 (7·0) |  | 24 (20·3) | 15 (7·4) |  | 15 (6·1) | 6 (9·4) |  | 9 (28·1) | 22 (15·7) |
| Coronary Heart Disease, N (%) | 9 (15·0) | 78 (13·0) |  | 24 (20·3) | 31 (15·3) |  | 30 (12·2) | 11 (17·2) |  | 7 (21·9) | 30 (21·4) |
| Cancer, N (%) | 11 (18·3) | 79 (13·2) |  | 25 (21·2) | 38 (18·7) |  | 29 (11·8) | 5 (7·8) |  | 6 (18·8) | 22 (15·7) |
| Chronic Kidney Disease, N (%) | 2 (3·3) | 25 (4·2) |  | 16 (13·6) | 5 (2·5) |  | 8 (3·3) | 0 (0·0) |  | 2 (6·2) | 11 (7·9) |
| Medication use for Hypertension, N (%) | 45 (75·0) | 317 (53·0) |  | 87 (73·7) | 123 (60·6) |  | 129 (52·4) | 38 (59·4) |  | 26 (81·2) | 95 (67·9) |
| Medication use for Diabetes, N (%) | 9 (15·0) | 75 (12·5) |  | 25 (21·2) | 25 (12·3) |  | 22 (8·9) | 3 (4·7) |  | 12 (37·5) | 18 (12·9) |
| Medication use for Dyslipidemia, N (%) | 27 (45·0) | 194 (32·4) |  | 44 (37·3) | 55 (27·1) |  | 82 (33·3) | 14 (21·9) |  | 18 (56·2) | 57 (40·7) |
| Alcohol habit, N (%) |  |  |  |  |  |  |  |  |  |  |  |
| Non-Drinker | 39 (65·0) | 350 (58·5) |  | 70 (59·3) | 118 (58·1) |  | 134 (54·5) | 40 (62·5) |  | 20 (62·5) | 107 (76·4) |
| Several Drinker | 14 (23·3) | 131 (21·9) |  | 25 (21·2) | 44 (21·7) |  | 67 (27·2) | 9 (14·1) |  | 8 (25·0) | 18 (12·9) |
| Daily drinker | 7 (11·7) | 108 (18·1) |  | 23 (19·5) | 37 (18·2) |  | 37 (15·0) | 14 (21·9) |  | 4 (12·5) | 15 (10·7) |
| Smoking habit, N (%) |  |  |  |  |  |  |  |  |  |  |  |
| Non-Smoker | 45 (75·0) | 485 (81·1) |  | 79 (66·9) | 156 (76·8) |  | 197 (80·1) | 49 (76·6) |  | 24 (75·0) | 108 (77·1) |
| Ex Smoker | 10 (16·7) | 87 (14·5) |  | 36 (30·5) | 35 (17·2) |  | 28 (11·4) | 13 (20·3) |  | 7 (21·9) | 29 (20·7) |
| Current smoker | 5 (8·3) | 17 (2·8) |  | 3 (2·5) | 8 (3·9) |  | 12 (4·9) | 1 (1·6) |  | 1 (3·1) | 3 (2·1) |
| Living alone, N (%) | 27 (45·0) | 168 (28·1) |  | 47 (39·8) | 68 (33·5) |  | 80 (32·5) | 15 (23·4) |  | 10 (31·2) | 65 (46·4) |

Abbreviation: SD, standard deviation; MCI, mild cognitive impairment.

# **Supplementary Table S3.**

**Performances of all developed prediction models in training and testing datasets.**

|  | **Reference models** | |  | **Voice biomarker models** | | | |
| --- | --- | --- | --- | --- | --- | --- | --- |
|  | **Age sex model** | **Age sex education model** |  | **Voice model** |  | **Age sex voice model** | **Age sex education voice model** |
| ***DNN*** |  |  |  |  |  |  |  |
| ***Training Dataset*** |  |  |  |  |  |  |  |
| ROC-AUC (95%CI) | 0·70 (0·66, 0·73) | 0·69 (0·65, 0·72) |  | 0·86 (0·83, 0·89) |  | 0·89 (0·87, 0·91) | 0·84 (0·81, 0·87) |
| Sensitivity (95%CI) | 0·82 (0·78, 0·86) | 0·74 (0·69, 0·79) |  | 0·67 (0·62, 0·72) |  | 0·80 (0·76, 0·85) | 0·73 (0·68, 0·78) |
| Specificity (95%CI) | 0·5 (0·46, 0·54) | 0·58 (0·55, 0·62) |  | 0·87 (0·85, 0·90) |  | 0·83 (0·79, 0·85) | 0·80 (0·77, 0·83) |
| Accuracy (95%CI) | 0·61 (0·57, 0·64) | 0·64 (0·61, 0·67) |  | 0·77 (0·74, 0·80) |  | 0·81 (0·79, 0·84) | 0·77 (0·74, 0·80) |
| Positive predictive value (95%CI) | 0·45 (0·41, 0·48) | 0·47 (0·42· 0·51) |  | 0·72 (0·67, 0·77) |  | 0·69 (0·64, 0·74) | 0·64 (0·59, 0·69) |
| Negative predictive value (95%CI) | 0·85 (0·81, 0·89) | 0·82 (0·79, 0·87) |  | 0·84 (0·82, 0·87) |  | 0·90 (0·87, 0·92) | 0·86 (0·83, 0·89) |
|  |  |  |  |  |  |  |  |
| ***Testing Dataset*** |  |  |  |  |  |  |  |
| ROC-AUC (95%CI) | 0·71 (0·66, 0·76) | 0·67 (0·62, 0·72) |  | 0·81 (0·77, 0·85) |  | 0·88 (0·85, 0·91) | 0·89 (0·86, 0·92) |
| Sensitivity (95%CI) | 0·90 (0·85, 0·94_ | 0·78 (0·72, 0·84) |  | 0·75 (0·69, 0·81) |  | 0·88 (0·83, 0·92) | 0·85 (0·80, 0·90) |
| Specificity (95%CI) | 0·52 (0·46, 0·58) | 0·59 (0·54, 0·65) |  | 0·83 (0·78, 0·87) |  | 0·75 (0·70, 0·80) | 0·76 (0·71, 0·81) |
| Accuracy (95%CI) | 0·68 (0·64, 0·72) | 0·67 (0·63, 0·72) |  | 0·79 (0·75, 0·83) |  | 0·81 (0·78, 0·85) | 0·81 (0·77, 0·84) |
| Positive predictive value (95%CI) | 0·58 (0·53, 0·63) | 0·58 (0·53, 0·64) |  | 0·76 (0·70, 0·82) |  | 0·72 (0·66, 0·77) | 0·72 (0·67, 0·78) |
| Negative predictive value (95%CI) | 0·87 (0·82, 0·92) | 0·79 (0·73, 0·84) |  | 0·82 (0·77, 0·87) |  | 0·89 (0·85, 0·93) | 0·87 (0·83, 0·91) |
|  |  |  |  |  |  |  |  |
| **XGBoost** |  |  |  |  |  |  |  |
| ***Training Dataset*** |  |  |  |  |  |  |  |
| ROC-AUC (95%CI) | 0·78 (0·74, 0·81) | 0·79 (0·76, 0·82) |  | 0·93 (0·91, 0·94) |  | 0·93 (0·91, 0·94) | 0·92 (0·91, 0·94) |
| Sensitivity (95%CI) | 0·63 (0·58, 0·69) | 0·63 (0·58, 0·68) |  | 0·84 (0·80, 0·88) |  | 0·84 (0·80, 0·88) | 0·82 (0·78, 0·86) |
| Specificity (95%CI) | 0·79 (0·76, 0·82) | 0·81 (0·78, 0·84) |  | 0·85 (0·83, 0·88) |  | 0·85 (0·83, 0·88) | 0·85 (0·82, 0·88) |
| Accuracy (95%CI) | 0·74 (0·71, 0·77) | 0·75 (0·72, 0·78) |  | 0·85 (0·83, 0·87) |  | 0·85 (0·83, 0·87) | 0·84 (0·82, 0·87) |
| Positive predictive value (95%CI) | 0·60 (0·54, 0·65) | 0·62 (0·56, 0·67) |  | 0·74 (0·68, 0·78) |  | 0·74 (0·68, 0·78) | 0·73 (0·68, 0·77) |
| Negative predictive value (95%CI) | 0·82 (0·78, 0·84) | 0·82 (0·79, 0·84) |  | 0·92 (0·90, 0·94) |  | 0·92 (0·90, 0·94) | 0·91 (0·88, 0·93) |
|  |  |  |  |  |  |  |  |
| ***Testing Dataset*** |  |  |  |  |  |  |  |
| ROC-AUC (95%CI) | 0·80 (0·76, 0·84) | 0·78 (0·73, 0·82) |  | 0·81 (0·77, 0·85) |  | 0·88 (0·85, 0·91) | 0·88 (0·85, 0·91) |
| Sensitivity (95%CI) | 0·74 (0·67, 0·79) | 0·68 (0·61, 0·74) |  | 0·72 (0·66, 0·78) |  | 0·80 (0·75, 0·86) | 0·80 (0·75, 0·86) |
| Specificity (95%CI) | 0·76 (0·71, 0·81) | 0·76 (0·71, 0·81) |  | 0·78 (0·73, 0·83) |  | 0·79 (0·74, 0·84) | 0·79 (0·74, 0·84) |
| Accuracy (95%CI) | 0·75 (0·71, 0·79) | 0·72 (0·68, 0·77) |  | 0·76 (0·72, 0·79) |  | 0·80 (0·76, 0·83) | 0·80 (0·76, 0·83) |
| Positive predictive value (95%CI) | 0·69 (0·63, 0·75) | 0·67 (0·61, 0·74) |  | 0·71 (0·64, 0·77) |  | 0·74 (0·69, 0·79) | 0·74 (0·69, 0·79) |
| Negative predictive value (95%CI) | 0·80 (0·74, 0·84) | 0·76 (0·71, 0·81) |  | 0·79 (0·74, 0·84) |  | 0·85 (0·80· 0·89) | 0·85 (0·80· 0·89) |

Abbreviation: ROC-AUC, receiver operating characteristic curves and area under the curves; CI, confidence interval; DNN, Deep Neural Network; XGBoost, eXtreme Gradient Boosting decision tree algorithm.

CIs were obtained from computing 1000 bootstrap sets.

# **Supplementary Table S4.**

**AUCs (95% CIs) according to subgroups of age, sex, or education in voice biomarker models in training and testing datasets**

|  | **Voice model** |  | **Age sex voice model** | **Age sex education voice model** |
| --- | --- | --- | --- | --- |
| ***ROC-AUC (95%CI) in Training Dataset*** | | | |  |
| **Age** |  |  |  |  |
| <75 years old | 0·86 (0·78, 0·92) |  | 0·87 (0·80, 0·93) | 0·78 (0·68, 0·87) |
| >=75 years old | 0·86 (0·83, 0·89) |  | 0·88 (0·86, 0·91) | 0·82 (0·79, 0·85) |
| **Sex** |  |  |  |  |
| Men | 0·85 (0·81, 0·89) |  | 0·88 (0·85, 0·92) | 0·81 (0·76, 0·85) |
| Women | 0·86 (0·83, 0·89) |  | 0·89 (0·87, 0·92) | 0·85 (0·82, 0·88) |
| **Education** |  |  |  |  |
| <=12 years | 0·86 (0·83, 0·89) |  | 0·89 (0·86, 0·91) | 0·83 (0·80, 0·85) |
| >12 years | 0·89 (0·84, 0·95) |  | 0·92 (0·87, 0·96) | 0·88 (0·82, 0·94) |
| **Data source** |  |  |  |  |
| Survey in Nobeoka | 0·84 (0·81, 0·87) |  | 0·88 (0·85, 0·90) | 0·81 (0·77, 0·84) |
| Survey in Kobe | 0·87 (0·82, 0·92) |  | 0·84 (0·78, 0·90) | 0·77 (0·70, 0·85) |
|  |  |  |  |  |
| ***ROC-AUC (95%CI) in Testing Dataset*** | | | |  |
| **Age** |  |  |  |  |
| <75 years old | 0·76 (0·56, 0·93) |  | 0·70 (0·52, 0·87) | 0·78 (0·65, 0·90) |
| >=75 years old | 0·81 (0·76, 0·85) |  | 0·87 (0·83, 0·90) | 0·88 (0·84, 0·91) |
| **Sex** |  |  |  |  |
| Men | 0·75 (0·67, 0·82) |  | 0·82 (0·75, 0·88) | 0·81 (0·73, 0·87) |
| Women | 0·84 (0·79, 0·88) |  | 0·91 (0·87, 0·94) | 0·92 (0·89, 0·95) |
| **Education** |  |  |  |  |
| <=12 years | 0·82 (0·77, 0·86) |  | 0·89 (0·85, 0·92) | 0·90 (0·86, 0·92) |
| >12 years | 0·79 (0·69, 0·88) |  | 0·88 (0·81, 0·95) | 0·85 (0·78, 0·93) |
| **Data source** |  |  |  |  |
| Survey in Nobeoka | 0·61 (0·53, 0·69) |  | 0·80 (0·74, 0·85) | 0·80 (0·74, 0·84) |
| Survey in Kobe | 0·62 (0·51, 0·73) |  | 0·80 (0·71, 0·88) | 0·79 (0·70, 0·87) |

Abbreviation: ROC-AUC, receiver operating characteristic curves and area under the curves; CI, confidence interval.

CIs were obtained from computing 1000 bootstrap sets.

We performed 10 bootstrap sets for the group ‘education>16 years’ since the sample size is small while we performed 1000 bootstraps for other groups.

# **Supplemental Figure S1.**

Flow chart illustrating how the study participants were divided into the training and testing datasets

# **Supplemental Figure S2.**

**Receiver operating characteristic curves and their AUCs for prediction performance of the developed cognitive impairment prediction models based on DNN**

Abbreviations: AUC, Area under the curves.

# **Supplementary Figure S3.**

**For voice model, two-dimensional visualisations of predictor variables from the proximity matrix of all participants using supervised UMAP, coloured by cognitive impairment and healthy observed status, in the testing data**

Abbreviations: UMAP, uniform a method of manifold approximation and projection.

Red dots show cognitive impairment individuals, and green dots show non-cognitive impairment individuals.


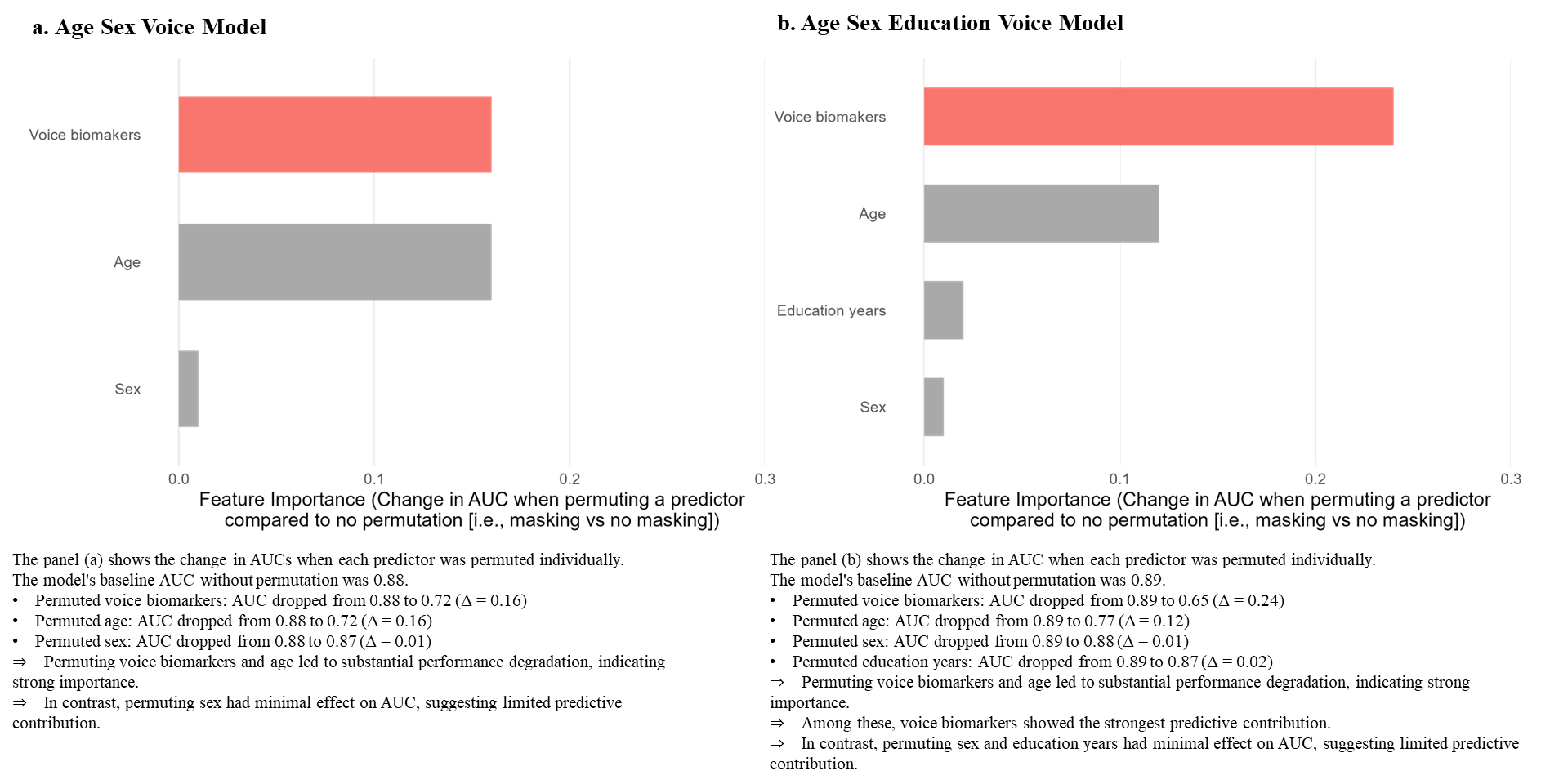


# **Supplementary Figure S4.**

**Feature importance estimated by grouped permutation feature importance for age sex voice model and age sex education voice model in the testing dataset**
Abbreviations: AUC, Area under the curves.
