# Supplementary material for: Developing and testing AI-based voice biomarker models to detect cognitive impairment among community dwelling adults: a cross-sectional study in Japan
Source: Lancet Reg Health West Pac. 2025 Jun 12;59:101598. doi: 10.1016/j.lanwpc.2025.101598 (PMC12266181; doi:10.1016/j.lanwpc.2025.101598)
Supplement: Translated abstract [file mmc2.docx]

**Editor disclaimer:** This translation in Japanese was submitted by the authors and we reproduce it as supplied. It has not been peer reviewed. Our editorial processes have only been applied to the original abstract in English, which should serve as reference for this manuscript.

**背景：** 軽度認知機能障害（MCI）は声のパターンやテンポに変化が生じると報告があり、音声は認知機能障害のバイオマーカーになる可能性がある。人工知能（AI）を用いることにより音声バイオマーカーを予測の特徴量として扱うことが可能となるため、早期に非侵襲かつ費用対効果の高い認知機能障害の識別が可能となると考えた。本研究では、日本の地域在住高齢者の非構造的な会話の声からAIで抽出した音声バイオマーカーを用いて、高精度な認知機能障害を識別する予測モデルを開発し、開発で使用していないテストデータを用いた検証の実施を目的とした。

**方法：**本研究のデザインは横断研究で、１，４６１名の地域在住高齢者を対象とした。認知機能の評価はMCI screenのMemory Performance Index (MPI)スコア（97%のAccuracyでMCIを識別）を用いた。３分間のオープンクエスチョンインタビューを行い音声データを収集し、音声generatorであるWav2Vec2を用いて音声バイオマーカーを抽出した。この音声バイオマーカーは音響特徴および韻律特徴に基づいており、個人の音声情報を512次元のベクトルとして表現している。そのほかの予測モデルの共変量を年齢、性別、教育歴とした。認知機能障害予測モデル構築は機械学習アルゴリズムのextreme gradient boosting decision tree algorithm とdeep neural network modelを979名の学習データで実施し、予測精度は学習で使用しなかった482名のテストデータで検証し、area under the curves (AUCs)を算出した。

**結果：**対象者のうち、女性967名(66·2%)、認知機能障害の者は526名(36·0%)、平均年齢（標準偏差）が79·5 (6·3)歳、平均教育年数が11·6 (2·2)年であった。認知機能障害の予測精度は音声バイオマーカーを追加することでAUC(95%信頼区間[CI])が有意に精度向上した。具体的には、AUC(95%CI)は年齢と性別のモデルで0·80 (0·76, 0·84) から 0·88 (0·84, 0·91)の精度向上、年齢、性別、教育歴のモデルで0·78 (0·73, 0·82) から 0·89 (0·86, 0·92)精度向上であった（どちらもDeLong testでp<0·0001を示した）。

**解釈：** 本研究において我々は認知機能障害を高精度に予測した(AUC = 0·89)。音声バイオマーカーを用いることで、予測精度の向上とスクリーニング時間の大幅な短縮に貢献可能と考える。
